# Platelet mitochondrial complex I and IV activities are not reliable stratification biomarkers in Parkinson's disease

**DOI:** 10.1177/1877718X251365253

**Published:** 2025-08-14

**Authors:** Simon Ulvenes Kverneng, Sepideh Mostafavi, Yana Mikhaleva, Gard Aasmund Skulstad Johanson, Haakon Berven, Katarina Lundervold, Geir Olve Skeie, Erika Sheard, Mona Søgnen, Solveig Af Geijerstam, Therese Vetås, Michele Brischigliaro, Erika Fernandez-Vizarra, Yamila N Torres Cleuren, Christian Dölle, Charalampos Tzoulis

**Affiliations:** 1 Neuro-SysMed, Department of Neurology, Haukeland University Hospital, Bergen, Norway; 2 Department of Clinical Medicine, University of Bergen, Bergen, Norway; 3 K.G. Jebsen Center for Translational Research in Parkinson's disease, University of Bergen, Bergen, Norway; 4 Department of Biomedical Sciences, University of Padova, Padova, Italy; 5 Veneto Institute of Molecular Medicine, Padova, Italy

**Keywords:** Parkinson's disease, biomarkers, mitochondria, blood platelets, skeletal muscle

## Abstract

**Background:**

Mitochondrial dysfunction, particularly complex I (CI) deficiency, is considered an integral feature of Parkinson's disease (PD). However, recent findings indicate that widespread neuronal CI deficiency in the brain is only present in a subpopulation of 20–30% of cases. This stratification may be relevant for selecting participants for clinical trials, emphasizing the need for clinically applicable biomarkers. We previously reported CI deficiency in skeletal muscle biopsies of a subpopulation of persons with PD (PwPs), suggesting potential for mitochondrial stratification using extra-neural tissues. Platelets are another tissue previously reported to exhibit mitochondrial respiratory defects in PD. However, studies have generally involved small sample sizes and reported variable results.

**Objective:**

To determine whether platelets exhibit impaired mitochondrial respiratory chain complex activity in PwPs, or in a subpopulation of PwPs.

**Methods:**

Using spectrophotometric activity assays, we assessed CI and complex IV (CIV) activities in platelet samples from 61 PwPs and 31 neurologically healthy controls from a well-characterized prospective cohort. The correlation between activities measured in platelets and skeletal muscle was also explored in 51 of the same individuals.

**Results:**

Platelet CI and CIV activities showed no difference between PwPs and controls at the group level, nor evidence of a subgroup with deficiency of either complex. There was no correlation between complex activities in platelet samples and skeletal muscle biopsies from the same individuals.

**Conclusions:**

Based on these results, we propose that platelet CI or CIV activities are not sensitive markers of mitochondrial dysfunction in PD.

## Plain language summary

Dysfunction of the mitochondria, the cells’ powerhouses, plays an important role in Parkinson's disease (PD). However, recent findings suggest that this dysfunction may be more pronounced in some persons with PD (PwPs) than others. Analyses in tissue samples from autopsy have shown that approximately 20–30% of PwPs harbor widespread mitochondrial dysfunction in their brain. These individuals may be more susceptible to the effects of mitochondria-targeting treatments. Therefore, detecting these individuals during life would provide them with the opportunity to participate in specific clinical trials. Research by us and others has shown that it may be possible to identify PwPs with mitochondrial dysfunction by examining muscle biopsies. Since obtaining these samples requires an invasive procedure associated with significant discomfort, we sought to identify less invasive tests. Previous research has indicated that mitochondrial function may be impaired in platelets from PwPs, but findings have been variable and conflicting. In this work, we examined mitochondrial function in platelets from 61 PwPs and 31 neurologically healthy individuals. We found no evidence supporting that mitochondrial function is impaired in platelets of PwPs, nor any evidence of a subgroup with mitochondrial impairment, similar to those observed in brain and muscle tissue. These results indicate that platelets are not an optimal medium for identifying mitochondrial biomarkers in PD.

## Introduction

Parkinson's disease (PD) is the most common neurodegenerative movement disorder and one of the most rapidly growing causes of neurological disability.^1,2^ Current treatments for PD are purely symptomatic and make no impact on disease progression.^3,4^ A major obstacle preventing mechanistic and therapeutic breakthroughs in PD lies in its unresolved biological diversity.^5–7^ A growing body of clinical, neuropathological, and molecular evidence suggest that biological subtypes of PD, beyond the known monogenic forms, may exist, each driven by different molecular mechanisms and, possibly, susceptible to different therapeutic strategies.^4,8,9^ Due to this variability, a successful treatment strategy for PD necessitates the development of biomarkers capable of stratifying affected individuals into biologically meaningful subgroups, enabling targeted therapeutic interventions.^4^

PD is strongly associated with mitochondrial dysfunction characterized by respiratory complex I (CI) deficiency.^10–12^ However, recent findings by our group indicate that the extent and distribution of neuronal CI deficiency are highly variable and stratify persons with PD (PwPs) into subgroups. One subgroup, comprising approximately 20–30% of PwPs, is characterized by widespread neuronal CI deficiency across multiple brain regions. We have termed this subgroup PD with CI deficiency. In contrast, the remaining 70–80% of PwPs show no evidence of CI deficiency outside the dopaminergic neurons of the substantia nigra.^13^ A similar trend is observed in skeletal muscle biopsies, where only a minor subpopulation of PwPs exhibit functional CI deficiency compared to age-matched controls.^14^ Further support for a mitochondrial subtype of PD comes from studies employing polygenic risk scores^15^ and assessment of mtDNA lesions in blood cells.^16^ While the overlap between the subgroups identified in these studies is yet to be determined, their collective findings support the existence of a distinct mitochondrial PD subtype, warranting further investigation.

Previous studies have detected mitochondrial respiratory chain (MRC) deficiencies, particularly of CI, in platelets of PwPs. However, results are conflicting, with a significant proportion of studies on MRC function in platelets reporting no difference between PwPs and healthy controls (reviewed by Subrahmanian and LaVoie).^17^ Furthermore, several of the studies that report CI deficiency in platelets show considerable overlap between the PD and control groups,^18–21^ which may be suggestive of a subgroup of PD cases with CI deficiency, similar to what we have observed in skeletal muscle and brain.^13,14^ This raises the possibility that the PD subgroup with CI deficiency can be discerned using platelet samples.

To address this question, we assessed the enzymatic activities of MRC CI and complex IV (CIV) in platelet samples from 61 individuals with clinically verified PD and 31 neurologically healthy controls collected from our longitudinal STRAT-PARK cohort.^22^ We also explored the correlation between CI and CIV activity in platelet samples and skeletal muscle samples, using data from our previous analysis of MRC activity in skeletal muscle biopsies from a subset of the same individuals.^14^ Our study aimed to determine the extent of mitochondrial dysfunction in platelets in PD and clarify whether PD can be stratified according to CI or CIV activity using easily accessible platelet samples.

## Methods

### Cohort characteristics

Platelet samples were collected at the Neuro-SysMed Center, Haukeland University Hospital (HUS), Bergen, Norway, during the baseline visit of 61 PwPs and 31 neurologically healthy controls participating in the STRAT-PARK cohort.^22^ PwPs fulfilled the Movement Disorder Society Clinical Diagnostic Criteria^23^ for established (*n* = 59) or probable (*n* = 2) PD, and showed evidence of nigrostriatal degeneration on ¹²³I-ioflupane single-photon emission computed tomography (SPECT; DaTscan). Medical history, complete neurological examination, and cognitive screening using the Montreal Cognitive Assessment^24^ were available from all participants. Information on the duration of motor symptoms and the Movement Disorder Society Unified Parkinson's Disease Rating Scale (MDS-UPDRS) score parts I-IV^25^ was available from all PwPs. Clinical and demographic features of the study cohort are provided in Supplemental Table 1. The study was conducted in full accordance with the International Council for Harmonisation (ICH) E6 guideline for Good Clinical Practice (GCP) and the principles of the Declaration of Helsinki, and the laws and regulations of Norway, including the General Data Protection Regulation. The study was approved by the Regional Committee for Medical and Health Research Ethics, Western Norway (STRAT-PARK, REK 74985). Written informed consent was obtained from all participants.

### Platelet isolation and cryopreservation

Whole blood was collected in a 9 mL VACUETTE^®^ tube ACD-A (acid citrate dextrose). After centrifugation at room temperature at 200 × g for 20 min without brake, 2375 μL of the top straw-colored layer was transferred to a new 8 mL tube. 125 μL of dimethyl sulfoxide (DMSO) was added and mixed thoroughly and the resulting platelet-rich plasma was aliquoted into four tubes of 500 μL. The aliquots were stored temporarily at −80°C for at least 4 h in a Mr Frosty^TM^ Freezing container (Thermo Scientific) before long term storage at −80°C.

### Preparation of mitochondria-enriched pellets

A phosphate-buffered saline (PBS) and ethylenediaminetetraacetic acid (EDTA) mix was prepared by mixing 5 mL of PBS and 10 μL of 0.5 M EDTA pH 8 per sample. From each study subject, three aliquots of 500 μL platelet-rich plasma were thawed in a water bath at 37°C and placed on ice. The aliquots were then pooled into a single 15 mL Falcon tube and mixed with of 3.5 mL of PBS/EDTA. The tubes were then centrifuged at 4°C for 10 min at 2000 × g with brake. The resulting supernatant was discarded, and the pellet was resuspended in 500 μL of PBS/EDTA before transfer to a 2.0 mL Sorenson^TM^ Dolphin microcentrifuge tube (Z717533-1000EA). The tubes were then centrifuged at 4°C for 10 min at 3000 × g with brake and the supernatant was discarded. The pellet was resuspended in 200 μL of buffer A (250 mM sucrose and 20 mM 3-(N-morpholino)propanesulfonic acid (MOPS) adjusted to pH 7.4 with potassium hydroxide (KOH)) and 200 μL of 0.2 mg/mL digitonin solution was added. Samples were kept on ice for 5 min before centrifugation at 5000 × g at 4°C for 5 min with brake. The supernatant, containing the cytosolic fraction, was discarded and the pellet was resuspended in 600 μL of buffer B (250 mM sucrose, 20 mM MOPS KOH pH 7.4, 1 mM EDTA pH 8.0) and kept on ice for 5 min. After a final centrifugation at 10 000 × g at 4°C for 5 min with brake, the supernatant was discarded, and mitochondria-enriched pellets were frozen in dry ice and stored at −80°C.

### Mitochondrial respiratory chain enzymatic activity measurements in platelet samples

Before activity measurements, mitochondria-enriched pellets were resuspended in 270 μL KP buffer (10 mM potassium phosphate, pH 7.4), snap-frozen in liquid nitrogen, and thawed at 37°C. This freeze-thaw cycle was repeated three times. Enzymatic activity measurements of complex I (CI), complex IV (CIV), and citrate synthase were carried out as previously described.^26^ Samples were assayed in technical triplicates using a 96-well plate and a TECAN Spark® microplate reader. Measurements were carried out in a total of three batches. Briefly, the activity of CI was measured by following the decrease in absorbance at λ = 340 nm caused by the oxidation of reduced nicotinamide adenine dinucleotide (NADH). 56 μL of the mitochondrial lysate was added to 144 μL of a reaction mixture containing the following components: 100 μL of 40 mM KP buffer (pH 8.0), 20 μL of 2 mM NADH, 4 μL of 50 mM NaN_3_, 20 μL of 10 mM bovine serum albumin in 10 mM EDTA (pH 7.4). After gentle mixing by pipetting, the reaction was pre-incubated for 2 min before measuring baseline change of absorbance. 10 μL of 1 mM coenzyme Q1 (in 10% EtOH) was added to each well to start the reaction, and change of absorbance was recorded for 2 min at 30°C. Next, change of absorbance was recorded again after addition of 4 μL of 0.25 mM rotenone (prepared from a stock solution of 2.5 mM rotenone in 96% EtOH:DMSO (1:1) diluted to 0.25 mM rotenone using 25% EtOH) to each well. The activity of CIV was measured by following the decrease in absorbance at λ = 550 nm caused by the oxidation of reduced cytochrome c. Baseline change of absorbance was measured in 186 μL reduced cytochrome c solution (100 μM in 50 mM KP buffer pH 7.0). 14 μL of mitochondrial lysate was added to start the reaction, and change of absorbance was recorded for 2 min at 37°C. Finally, the activity of citrate synthase was measured indirectly by following the increase in absorbance at λ = 412 nm at 30°C caused by the formation of thionitrobenzoate (TNB) due to the reaction of 5,5-Dithiobis(2-nitrobenzoic acid) (DTNB) with free coenzyme A produced by the activity of the enzyme. 10 μL of mitochondrial lysate was added to 180 μL of a reaction mix consisting of 20 μL of 750 mM tris(hydroxymethyl)aminomethane hydrochloride (Tris-HCl) buffer (pH 8.0), 20 μL of 1 mM DTNB, 4 μL of 5% Triton X-100, 10 μL of 8 mM Acetyl-CoA and 126 μL of ddH_2_O. After gentle mixing by pipetting, absorbance change was measured at baseline for 2 min. Then, 10 μL of 1.32 mg/ml oxaloacetate in ddH_2_O was added to each well to start the reaction and change of absorbance was recorded for 2 min at 30 °C. Protein concentration in the mitochondrial lysates were determined using Pierce^TM^ bicinchoninic acid (BCA) Protein Assay Kit (ThermoFisher. #23225), as per the manufacturer's instruction. Specific activity (SA) values were calculated using the following formula: $\frac{\left(\frac{\Delta A}{min}\;x\;mL\;of\;reaction\right)}{\left(\varepsilon \;x\;mL\;of\;sample\;x\;\frac{mg}{mL}\;of\;sample\;protein\right)}$ . For the calculation of CI activity, the molar extinction coefficient (ɛ) of 6.2 mM^−1^cm^−1^ was used for NADH; for the calculation of CIV activity, ɛ of 18.5 mM^−1^cm^−1^ was used for reduced cytochrome c; and for calculation of citrate synthase activity, ɛ of 13.8 mM^−1^cm^−1^ was used for TNB.

### Skeletal muscle biopsy and mitochondrial respiratory chain enzymatic activity measurements

Needle biopsies of the vastus lateralis muscle were collected from a subset of the subjects during the same study visit as the collection of platelet samples, using a Bard Magnum biopsy instrument (BD^©^, United States).^22^ Sample preparation and spectrophotometric measurement of MRC activity in these samples have been detailed previously.^14^ Briefly, 5–10 mg of each muscle biopsy was minced with a surgical scalpel and homogenized using a glass-glass Dounce homogenizer (15 manual strokes) in 20 volumes of Medium A (0.32 M sucrose, 10 mM Tris-HCl pH 7.4, 1 mM EDTA). The homogenate was centrifuged at 800 × g for 5 min at 4°C. The supernatant was collected, frozen at −80°C overnight, and subjected to two freeze-thaw cycles (thawing at 37°C and snap-freezing in liquid nitrogen) before analysis. The activities of MRC complexes I, II, III, and IV, as well as citrate synthase, were determined spectrophotometrically using 10–30 µl of mitochondria-enriched supernatant, in a total rection volume of 200 µl, as described.^14,26^

### Statistical analysis

For each biological sample, citrate synthase, CI, and CIV activities were assayed in technical triplicates whenever possible. The coefficient of variation was calculated for the triplicates, defined as the ratio of the standard deviation to the mean, expressed as a percentage. Triplicates displaying a coefficient of variation above 25% were inspected for outliers. In cases where removal of one outlier resulted in two remaining replicates that were no more than 25% different from their mean, the sample was kept in the dataset. In some cases, only technical duplicates were available due to limited sample material. In these instances, the sample was kept in the dataset if the replicates were no more than 25% different from their mean. Samples that did not fulfill these criteria were omitted from further statistical analysis. The activities of CI and CIV were normalized to citrate synthase activity to account for potential differences in mitochondrial content. Data were assessed for normality using the Shapiro-Wilk test and for equality of variance using Levene's test. Univariate between-group comparisons were performed using either an independent Student's *t*-test or a Wilcoxon rank-sum test with continuity correction, depending on data normality. Correlation between two continuous variables was assessed using either Pearson's product-moment correlation or Spearman's rank correlation, depending on data normality. Association between two categorical variables was tested using the Fisher's exact test. Further analyses of MRC activity data were performed using multiple linear regression models. Citrate synthase-normalized CI- and CIV-activities, and citrate synthase activity alone, were log-transformed to improve model fits. Disease status, age, sex, smoking status, and measurement batch were used as independent variables. Multiple linear regression analyses of citrate synthase-normalized CI and CIV-activities were repeated with the omission of active smokers. Variance inflation factors were used to rule out collinearity in the models.

The comparison of citrate synthase-normalized CI and CIV-activities in platelet samples and skeletal muscle samples from the same individuals was performed with and without adjusting the datasets for batch effects. Adjustment for batch effects was achieved by linear regression models using only measurement batch as the independent variable and the activity measurement as the dependent variable (e.g., citrate synthase-normalized CI-activity). In all instances, the dependent variable was log transformed improve model fits. The residuals (i.e., variation in the data not explained by batch) were extracted and the intercept of the regression was re-added to maintain the original scale. The batch-adjusted datasets were used to compare citrate synthase-normalized CI and CIV-activities in platelet samples and skeletal muscle samples by correlation tests and by visual inspection of distributions.

All analyses were performed using R version 4.3.0 (R Core Team, 2023) in RStudio 2023.03.1 Build 446 (2009–2023 Posit Software, PBC). Plots were made using the ggplot2 package V3.4.4.^27^ Adjustment for batch effects was performed using the *adjust* function of the datawizard package V0.9.1.^28^

## Results

### No evidence of complex I or IV deficiency in PD platelets

**Figure 1.**
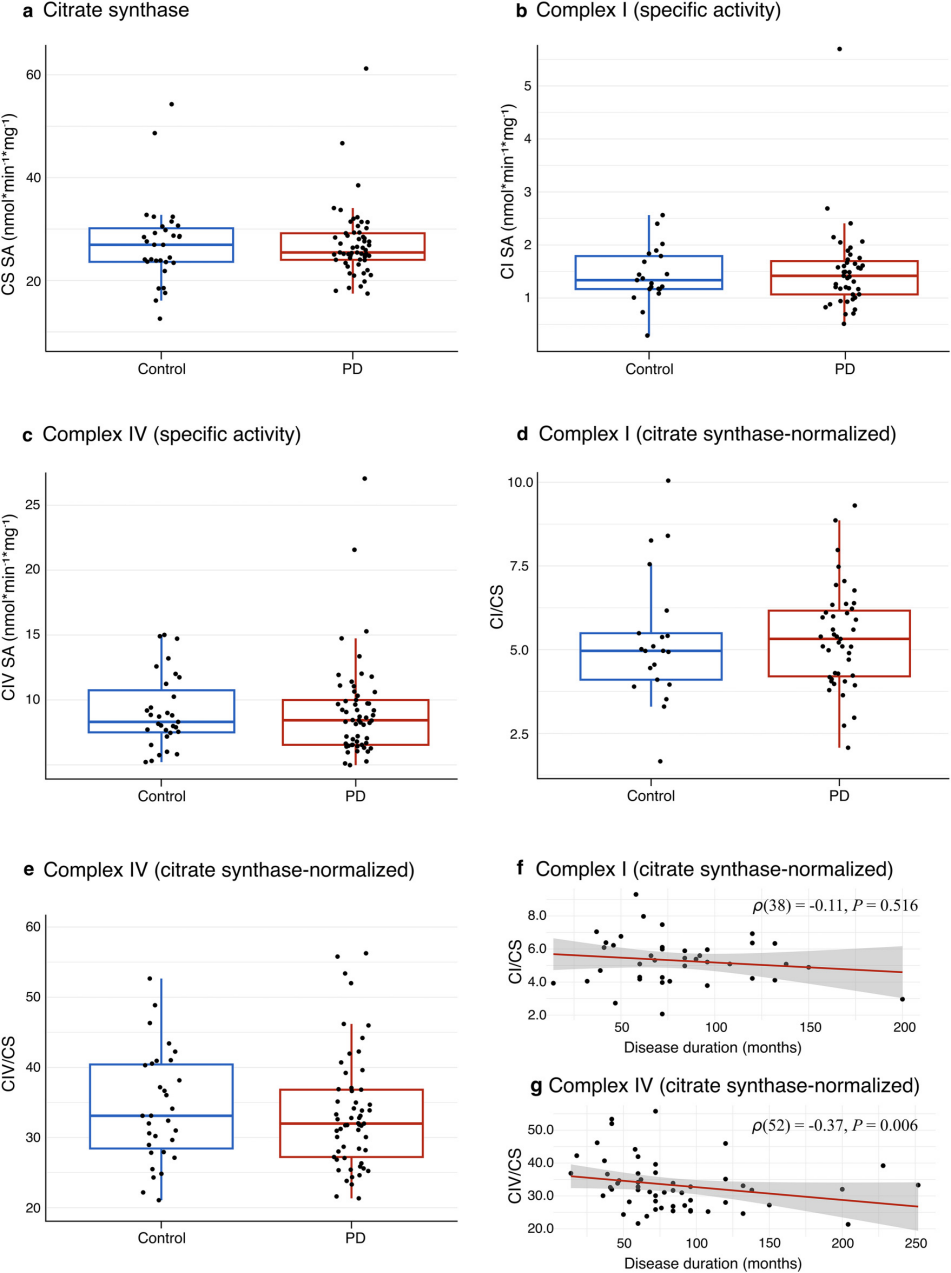
Citrate synthase, CI, and CIV activity measurements in platelet samples from PwPs and controls. Activities of citrate synthase, complex I (CI), and complex IV (CIV) in platelet samples from PwPs and controls. Boxes: median and interquartile range (IQR); whiskers: 1.5 x IQR from the lower and upper quartiles. Each dot represents one individual. (a) Specific activity of CS. (b) Specific activity of CI. (c) Specific activity of CIV. (d) Citrate synthase-normalized CI activity. (e) Citrate synthase-normalized CIV activity. (f-g) Correlation between duration of motor symptoms and citrate synthase-normalized CI activity (f) and citrate synthase-normalized CIV activity (g). The Spearman's correlation coefficient, along with the corresponding *P*-value, is shown. Current smokers (*n* = 3) have been removed in (f-g).

The specific enzymatic activities of CI, CIV, and citrate synthase were measured in mitochondria-enriched fractions prepared from platelet-rich plasma from 61 PwPs and 31 neurologically healthy controls. The demographic and clinical characteristics of the full study cohort are summarized in Supplemental Table 1, while Tables 1 and 2 provide stratified characteristics according to each analysis. There was a significantly larger female proportion in the control group, which was also slightly younger than the PD group. Analyses were run in technical replicates and the coefficient of variation was used to identify and exclude outliers (see Methods and Supplemental Data 1). Samples from four PwPs were omitted from statistical analysis due to highly variable citrate synthase activity in technical replicates, indicating poor sample quality. The resulting dataset consisted of 57 PwPs and 31 controls (Table 1). Specific citrate synthase activity was similar in the PD and control groups (Wilcoxon rank-sum test, *W* = 895, *P* = 0.924; Figure 1(a)), indicating similar mitochondrial mass. Furthermore, multiple linear regression analysis revealed no association between citrate synthase activity and disease status, age, sex, smoking status, or experimental batch (Table 3).

**Table 1.** Demographic and clinical characteristics of subjects included in CS and CIV activity analyses.

	Group		
Variable	PD (*n* = 57)	Control (*n* = 31)	*P*-value
Sex (male/female)	33/24	8/23	**0**.**007**^a^
Age (years)	69.1 ± 7.6	64.7 ± 10.0	**0**.**022**^b^
MDS diagnosis (est./prob.)	55/2	-	
Disease duration (years)	7.0 ± 4.1	-	
Motor phenotype	TD: 22	-	
	PIGD: 29		
	IND: 6		
MDS-UPDRS III score	28.2 ± 11.5	-	
Hoehn & Yahr score	2.1 ± 0.6	-	
MoCA score	24.5 ± 3.0	25.8 ± 2.4	0.067^c^

MDS diagnosis: PD diagnosis according to the Movement Disorder Society (MDS) Clinical Diagnostic Criteria for PD; est.: established; prob.: probable; Disease duration (years): duration of motor symptoms in years; TD: tremor dominant; PIGD: postural instability/gait difficulty; IND: indeterminate; MDS-UPDRS III: Movement Disorder Society Unified Parkinson's Disease Rating Scale part III; MoCA: Montreal Cognitive Assessment.

Age, disease duration, MDS-UPDRS III score, Hoehn & Yahr stage, and MoCA score are presented as mean ± standard deviation. Significant *P*-values are in bold.

^a^ Two-sided Fisher's exact test.

^b^ Student's *t*-test.

^c^ Wilcoxon rank-sum test.

**Table 2.** Demographic and clinical characteristics of subjects included in CI activity analyses.

	Group		
Variable	PD (*n* = 43)	Control (*n* = 21)	*P*-value
Sex (male/female)	26/17	5/16	**0**.**008**^a^
Age (years)	69.4 ± 7.1	63.8 ± 9.5	**0**.**010**^b^
MDS diagnosis (est./prob.)	42/1	-	
Disease duration (years)	6.7 ± 3.2	-	
Motor phenotype	TD: 18		
	PIGD: 20		
	IND: 5		
MDS-UPDRS III score	26.7 ± 10.6	-	
Hoehn & Yahr score	2.0 ± 0.5	-	
MoCA score	25.0 ± 3.0	26.6 ± 1.9	0.055^c^

MDS diagnosis: PD diagnosis according to the Movement Disorder Society (MDS) Clinical Diagnostic Criteria for PD; est.: established; prob.: probable; Disease duration (years): duration of motor symptoms in years; TD: tremor dominant; PIGD: postural instability/gait difficulty; IND: indeterminate; MDS-UPDRS III: Movement Disorder Society Unified Parkinson's Disease Rating Scale part III; MoCA: Montreal Cognitive Assessment.

Age, disease duration, MDS-UPDRS III score, Hoehn & Yahr stage, and MoCA score are presented as mean ± standard deviation. Significant *P*-values are in bold.

^a^ Two-sided Fisher's exact test.

^b^ Student's *t*-test.

^c^ Wilcoxon rank-sum test.

**Table 3.** Multiple linear regression models of enzymatic activity of CS, CI, and CIV in platelet samples from PwPs and controls.

	Dependent variable								
	log(CS)			log(CI/CS)			log(CIV/CS)		
Predictors	*B*	95% CI	*P*-value	*B*	95% CI	*P*-value	*B*	95% CI	*P*-value
Status (PD)	0.012	−0.038–0.061	0.634	−0.003	−0.082–0.076	0.933	−0.021	−0.068–0.026	0.383
Age	−0.002	−0.004–0.001	0.256	0.001	−0.003–0.006	0.516	0.003	0.001–0.006	**0.017**
Sex (Male)	0.007	−0.040–0.055	0.768	−0.005	−0.075–0.065	0.880	−0.004	−0.049–0.041	0.864
Smoking	−0.008	−0.115–0.099	0.881	0.047	−0.111–0.206	0.552	0.009	−0.093–0.110	0.867
Batch 1	Reference	-	-	-	-	-	-	-	-
Batch 2	1.5 × 10^−04^	−0.056–0.055	0.996	0.041	−0.048–0.131	0.361	−0.009	−0.061–0.044	0.745
Batch 3	−0.055	−0.111–0.001	0.053	−0.128	−0.203 – −0.053	**0.001**	−0.049	−0.101–0.004	0.071
Observations	88			64			88		
R^2^ / R^2^ adjusted	0.088 / 0.020			0.257 / 0.178			0.101 / 0.034		

CI: specific complex I activity; CIV: specific complex IV activity; CS: specific citrate synthase activity; *B*: regression coefficient (unstandardized); 95% CI: 95% confidence interval of the regression coefficient. Significant *P*-values are in bold. Nominal *P-*values are given.

The activities of CI and CIV were normalized to citrate synthase activity to account for potential differences in mitochondrial content. The citrate synthase-normalized activities of CI and CIV are henceforth referred to simply as “CI and CIV activity” unless otherwise stated. Specific CI activity measurements from 14 PwPs and 10 controls were omitted from further analysis due to high variability of technical replicates, indicating technical issues with these samples. The resulting dataset of CI activity measurements consisted of 43 PwPs and 21 controls (Table 2). There was no significant difference in platelet CI activity between PwPs and controls at the group level (Student's *t*-test, *t*(62) = −0.12, *P* = 0.902). Furthermore, the distribution of CI activity did not suggest the presence of a PD subgroup with deficiency (Figure 1(b) and (d)). In a multiple linear regression model, there were no significant associations between CI activity and disease status, age, sex, or smoking status (Table 3).

CIV activity measurements generally displayed less technical variability than CI activity measurements, resulting in a final dataset of CIV activity measurements from 57 PwPs and 31 neurologically healthy controls (Table 1). Similar to CI, there was no evidence of CIV activity deficiency observed in the PD group compared to controls (Wilcoxon rank-sum test, *W* = 953, *P* = 0.547) and the distribution of CIV activity was similar in the two groups (Figure 1(c) and (e)). Additionally, there were no significant associations between CIV activity and disease status, sex, or smoking status (Table 3). However, we observed a significant positive association between CIV activity and age (multiple linear regression model, *B* = 0.003, *P* = 0.017; Table 3). Repeating the multiple linear regression model separately in the PD and control groups revealed that the association between CIV activity and age was only present in the control group (Supplemental Table 2).

There were only four current smokers in the dataset, comprising three PwPs and one healthy control. As smoking has previously been reported to negatively influence CI activity in platelets^29^ and decrease CIV activity in other blood components, such as lymphocytes,^30,31^ the analyses were repeated without the active smokers and showed similar results (Supplemental Table 3).

### Platelet complex I and IV activities are not associated with phenotypical features in PD

We next explored associations between platelet CI or CIV activity and disease severity. Active smokers (*n* = 3) were omitted from these analyses. There was no significant correlation between disease duration, defined as months of motor symptoms, and CI activity (Spearman's correlation coefficient, *ρ*(38) = −0.11, *P* = 0.516; Figure 1(f)). Furthermore, in a multiple linear regression model with age, sex, measurement batch, disease duration, and MDS-UPDRS part III score as independent variables, there were no significant associations between either duration of motor symptoms or motor severity and CI activity (Supplemental Table 4). In contrast, there was a significant negative correlation between disease duration and CIV activity (Spearman's correlation coefficient, *ρ*(52) = −0.37, *P* = 0.006; Figure 1g). However, this association was not statistically significant in a multiple linear regression model that included the above-mentioned independent variables (Supplemental Table 4).

### No correlation between platelet and skeletal muscle CI and CIV activities

**Figure 2.**
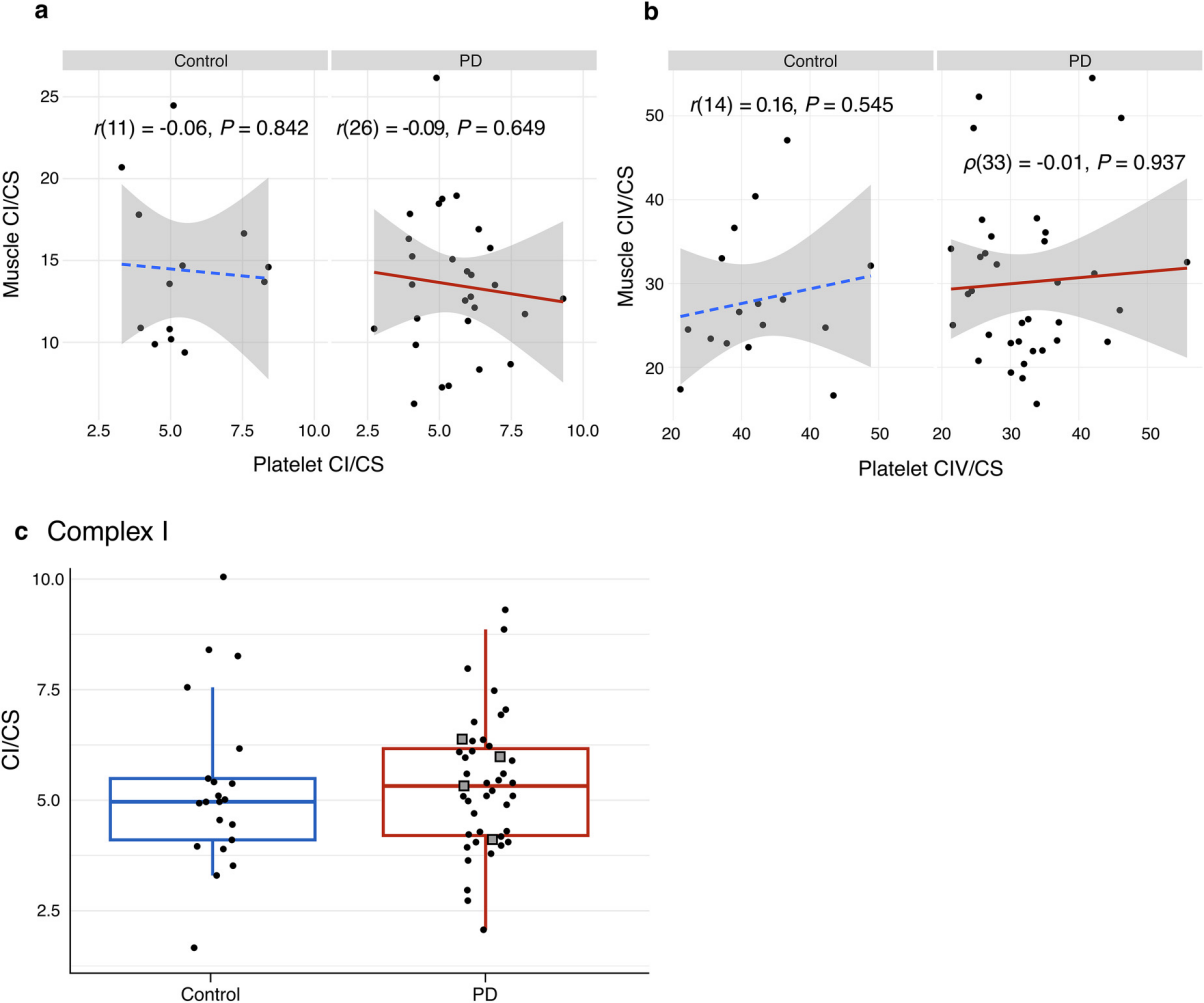
Comparison of platelet and skeletal muscle activities of CI and CIV. Scatter plots show correlation between platelet and skeletal muscle complex I (CI) (a) and complex IV (CIV) (b) activity within the PD group (solid lines) and the control group (dashed lines). CI and CIV activities are normalized to citrate synthase activity. Each dot represents one individual. The Spearman's and Pearson's correlation coefficients, along with their corresponding *P*-values, are shown. (c) Citrate synthase-normalized CI activity in platelets of PwPs and neurologically healthy controls. Boxes: median and interquartile range (IQR); whiskers: 1.5 x IQR from the lower and upper quartiles. Each dot represents one individual. Squares indicate PwPs who displayed skeletal muscle citrate synthase-normalized CI activity below the range of controls in a previous analysis.

MRC activity data from skeletal muscle biopsies collected at the same day as the platelets were available for 41 of the same individuals (28 PwPs and 13 controls) for CI and 51 of the same individuals (35 PwPs and 16 controls) for CIV.^14^ CI activity displayed no correlation between skeletal muscle and platelets in the PD or control group (Pearson's correlation coefficient, PD group: *r*(26) = −0.09, *P* = 0.649; control group: *r*(11) = −0.06, *P* = 0.842; Figure 2(a)). Similar results were observed for complex IV activity (PD group: Spearman's correlation coefficient, *ρ*(33) = −0.01, *P* = 0.937; control group: Pearson's correlation coefficient: *r*(14) = 0.16, *P* = 0.545; Figure 2(b)). Since the MRC activity assays for platelets and muscle were conducted at different times by different personnel, and in order to mitigate the impact of technical variability, both datasets were adjusted for the effects of experimental batch using linear regression (see Methods). Still, this did not reveal any significant correlations between platelets and skeletal muscle samples for any of the complexes (Supplemental Figure 1a, b). CI activity measurements in platelets were available from four of the PwPs who in a previous analysis displayed muscle CI activity below the range of healthy controls.^14^ As anticipated, given the lack of correlation between CI activity in platelets and muscle, these individuals did not show generally lower CI activity in platelets (Figure 2(c)). This was also the case after adjusting the platelet CI activity data for the effects of experimental batch (Supplemental Figure 1c).

## Discussion

In this population-based prospective cohort, we found no evidence of mitochondrial CI or CIV deficiency, nor altered mitochondrial content, in platelets of PwPs. Our findings are consistent with approximately half of the previous studies on platelets (8/15), but contrast with others that reported reduced activity of various MRC complexes, particularly CI, in PwPs.^17^

Several factors may underly these conflicting results. First, previous investigations into platelet MRC complex activities in PD have typically employed relatively small sample sizes, generally including fewer than 30 PwPs.^17^ The resulting limited power of these studies increases the likelihood of inconsistent and contradictory results. Second, variation in diagnostic accuracy may have contributed to the variability of previous results. Misdiagnosis of PD is not uncommon, particularly during early stages of the disease, and diagnostic accuracy tends to improve significantly with longitudinal follow up by movement disorders specialists,^32^ as practiced in systematic longitudinal cohorts like STRAT-PARK.^22^ Finally, differences in methodological approaches, including sample preparation, storage, and measurement, may also play a role.

While we cannot confidently exclude that previously observed discrepancies may reflect a subgroup effect, this is not supported by our data. The distribution of CI and CIV activity in the PD group was not consistent with the existence of a deficiency subgroup, in sharp contrast to our previous findings in the brain PD^13^ and skeletal muscle.^14^

The lack of findings in PD platelets may reflect their intrinsic biology, which differs markedly from that of skeletal myocytes and neurons. These cells exhibit high bioenergetic demands and are post-mitotic, allowing for the accumulation of mitochondrial damage over time.^33^ However, platelets are short-lived, with an average lifespan of only 7–10 days,^34^ have more modest bioenergetic requirements, at least when quiescent, and, while they respire, they usually rely more on glycolysis compared to muscle cells and neurons.^35^ Thus, the presence of functional CI deficiency in the brain^13^ and skeletal muscle,^14^ but not platelets, may simply reflect the fact that these tissues are generally more susceptible to mitochondrial dysfunction.

This is further corroborated by the fact that, similar to our findings, previous studies reported no correlation between mitochondrial respiration in platelets and skeletal muscle from the same individuals,^36,37^ indicating that mild mitochondrial abnormalities in skeletal muscle will not necessarily be detectable in platelets. This aligns with our results, showing no correlation between CI or CIV activities in skeletal muscle and platelets.

Sex differences in mitochondrial function have previously been reported to be minimal.^38^ Consistent with this assumption, we did not find any significant association between CI or CIV activity and sex in our data. Furthermore, we did not observe an association between CI activity in platelets and age, which is in line with previous studies in both PwPs and healthy controls.^18–20,39–41^ However, there was a significant positive association between CIV activity and age, which was present in the control group but not in the PD group. An increase of platelet CIV activity with age has previously been reported in healthy controls, possibly reflecting a compensatory mechanism to age-related mitochondrial dysfunction.^40^ The absence of a similar age-effect in the PD group may reflect a failure of such a compensatory mechanism, but is difficult to interpret as there was no group-level deficiency of CIV activity in PwPs compared to controls, despite adjusting for age in our models.

The main strengths of this study are its large sample size and the fact that the participants originate from a population-based, comprehensively characterized cohort of clinically verified PwPs and neurologically healthy controls.^22^ Apart from two cases of “probable PD”, all included cases had a diagnosis of “established PD” according to the Movement Disorder Society Clinical Diagnostic Criteria,^23^ and all diagnoses were supported by ¹²³I-ioflupane SPECT (DaTscan), confirming nigrostriatal denervation. Given the population-based nature of our cohort,^22^ our results are generalizable to the PD population of Western Norway.

Our study also has several limitations. The sensitivity of spectrophotometric activity assays may be insufficient to detect subtle differences in platelet CI and CIV activities between the PD and control group. Nevertheless, any such subtle differences would likely be too small to enable reliable clinical stratification. Furthermore, the PD and control groups were not fully matched for sex, with a larger proportion of females in the control group. This is related to the higher prevalence of PD in males,^42^ and most of the control individuals being spouses of the PwPs in our cohort.

In summary, our results do not support the presence of CI or CIV deficiency in platelets of PwPs – either at the group or subgroup level. Based on these results, we propose that platelet CI or CIV activities are not sensitive markers of mitochondrial dysfunction in PD.

## Supplemental Material

sj-docx-1-pkn-10.1177_1877718X251365253 - Supplemental material for Platelet mitochondrial complex I and IV activities are not reliable stratification biomarkers in Parkinson's disease Supplemental material, sj-docx-1-pkn-10.1177_1877718X251365253 for Platelet mitochondrial complex I and IV activities are not reliable stratification biomarkers in Parkinson's disease by Simon Ulvenes Kverneng, Sepideh Mostafavi, Yana Mikhaleva, Gard Aasmund Skulstad Johanson, Haakon Berven, Katarina Lundervold, Geir Olve Skeie, Erika Sheard, Mona Søgnen, Solveig Af Geijerstam, Therese Vetås, Michele Brischigliaro, Erika Fernandez-Vizarra, Yamila N Torres Cleuren, Christian Dölle and Charalampos Tzoulis in Journal of Parkinson's Disease

sj-docx-2-pkn-10.1177_1877718X251365253 - Supplemental material for Platelet mitochondrial complex I and IV activities are not reliable stratification biomarkers in Parkinson's disease Supplemental material, sj-docx-2-pkn-10.1177_1877718X251365253 for Platelet mitochondrial complex I and IV activities are not reliable stratification biomarkers in Parkinson's disease by Simon Ulvenes Kverneng, Sepideh Mostafavi, Yana Mikhaleva, Gard Aasmund Skulstad Johanson, Haakon Berven, Katarina Lundervold, Geir Olve Skeie, Erika Sheard, Mona Søgnen, Solveig Af Geijerstam, Therese Vetås, Michele Brischigliaro, Erika Fernandez-Vizarra, Yamila N Torres Cleuren, Christian Dölle and Charalampos Tzoulis in Journal of Parkinson's Disease

sj-xlsx-3-pkn-10.1177_1877718X251365253 - Supplemental material for Platelet mitochondrial complex I and IV activities are not reliable stratification biomarkers in Parkinson's disease Supplemental material, sj-xlsx-3-pkn-10.1177_1877718X251365253 for Platelet mitochondrial complex I and IV activities are not reliable stratification biomarkers in Parkinson's disease by Simon Ulvenes Kverneng, Sepideh Mostafavi, Yana Mikhaleva, Gard Aasmund Skulstad Johanson, Haakon Berven, Katarina Lundervold, Geir Olve Skeie, Erika Sheard, Mona Søgnen, Solveig Af Geijerstam, Therese Vetås, Michele Brischigliaro, Erika Fernandez-Vizarra, Yamila N Torres Cleuren, Christian Dölle and Charalampos Tzoulis in Journal of Parkinson's Disease
