# Supplementary material for: Platelet mitochondrial complex I and IV activities are not reliable stratification biomarkers in Parkinson's disease
Source: J Parkinsons Dis. 2025 Aug 14;15(7):1218–28. doi: 10.1177/1877718X251365253 (PMC13347526; doi:10.1177/1877718X251365253)
Supplement: sj-docx-2-pkn-10.1177_1877718X251365253 - Supplemental material for Platelet mitochondrial complex I and IV activities are not reliable stratification biomarkers in Parkinson's disease [file sj-docx-2-pkn-10.1177_1877718X251365253.docx]

**Supplementary information for**

**Platelet mitochondrial complex I and IV activities are not reliable stratification biomarkers in Parkinson’s disease**

Simon Ulvenes Kverneng *et al.*

Corresponding author: Charalampos Tzoulis
E-mail: [charalampos.tzoulis@uib.no](mailto:charalampos.tzoulis@uib.no)

E-mail-2: [charalampos.tzoulis@helse-bergen.no](mailto:charalampos.tzoulis@helse-bergen.no)

**This PDF file includes:**
Supplementary Fig. 1

Supplementary Tables 1 to 4

**
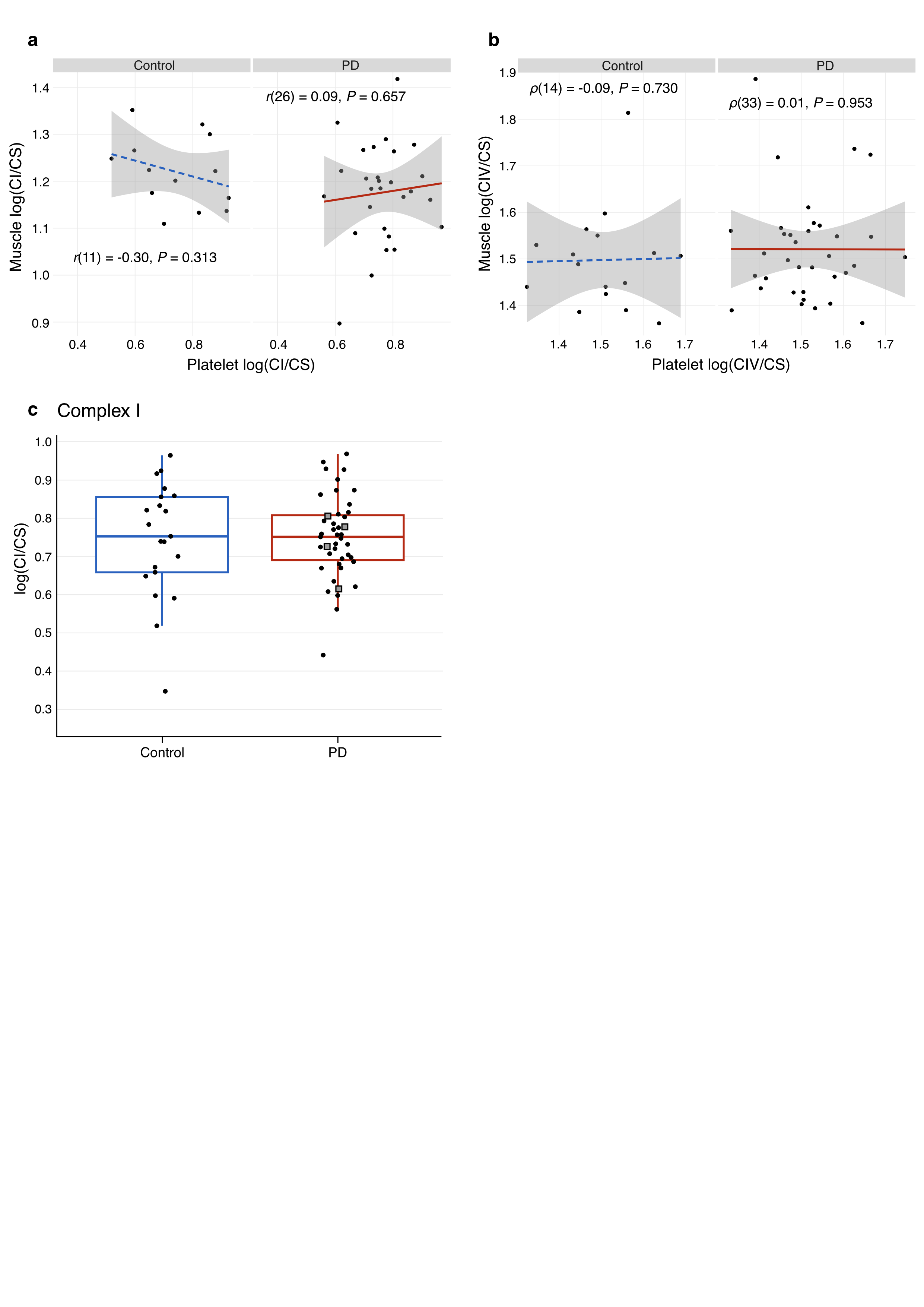
Supplementary Fig. 1. Comparison of platelet and skeletal muscle activities of CI and CIV with correction for batch effects.**

Scatter plots show correlation between platelet and skeletal muscle CI **(a)** and CIV **(b)** activity within the PD and control group. CI and CIV activities are normalized to citrate synthase activity. Each dot represents one individual. The platelet activity data and skeletal muscle activity data have been adjusted for the effect of measurement batch by regressing out this variable (see Methods section). **(c)** Activity of CI normalized to citrate synthase in platelets of PwPs and neurologically healthy controls. Boxes: median and interquartile range (IQR); whiskers: 1.5 x IQR from the lower and upper quartiles. Each dot represents one individual. Squares indicate PwPs who displayed skeletal muscle citrate synthase-normalized CI activity below the range of controls in a previous analysis. The platelet activity data have been adjusted for the effect of measurement batch by regressing out this variable (see Methods section).

| **Supplementary Table 1: Demographic and clinical characteristics of the study cohort** | | | |
| --- | --- | --- | --- |
|  | Group | |  |
| Variable | PD (*n* = 61) | Control (*n* = 31) | *P*-value |
| Sex (male/female) | 36/25 | 8/23 | **0.004^a^** |
| Age (years) | 68.7 ± 7.8 | 64.7 ± 10.0 | **0.037^b^** |
| MDS diagnosis (established/probable) | 59/2 | - | - |
| Disease duration (years) | 6.8 ± 4.1 | - | - |
| Motor phenotype: | TD: 24 PIGD: 31 IND: 6 | - | - |
| MDS-UPDRS III score | 28.0 ± 11.2 |  |  |
| Hoehn & Yahr stage | 2.1 ± 0.5 |  |  |
| MoCA score | 24.7 ± 3.1 | 25.8 ± 2.4 | 0.085^b^ |
| MDS diagnosis: PD diagnosis according to the Movement Disorder Society (MDS) Clinical Diagnostic Criteria for PD; Disease duration (years): duration of motor symptoms in years; TD: tremor dominant; PIGD: postural instability/gait difficulty; IND: indeterminate; MDS-UPDRS III: Movement Disorder Society Unified Parkinson’s Disease Rating Scale part III; MoCA: Montreal Cognitive Assessment.  Age, disease duration, MDS-UPDRS III score, Hoehn & Yahr stage, and MoCA score are presented as mean ± standard deviation. Significant *P*-values are in bold. | | | |
| ^a^Two-sided Fisher’s exact test  ^b^Student’s *t*-test | | |  |

| **Supplementary Table 2. Multiple linear regression model of CIV activity grouped by disease status.** | | | | | | |
| --- | --- | --- | --- | --- | --- | --- |
|  | *Dependent variable* | | | | | |
|  | **PD  log(CIV/CS)** | | | **Control log(CIV/CS)** | | |
| Predictors | *B* | 95 % CI | *P*-value | *B* | 95% CI | *P*-value |
| Age | 0.001 | -0.002 – 0.005 | 0.489 | 0.006 | 0.002 – 0.010 | **0.003** |
| Sex (Male) | 0.003 | -0.052 – 0.059 | 0.902 | -0.041 | -0.125 – 0.044 | 0.330 |
| Smoking | 0.026 | -0.098 – 0.151 | 0.670 | -0.075 | -0.272 – 0.121 | 0.437 |
| Batch 1 | Ref. | - | - | - | - | - |
| Batch 2 | -0.016 | -0.089 – 0.056 | 0.651 | 0.026 | -0.057 – 0.110 | 0.522 |
| Batch 3 | -0.065 | -0.136 – 0.005 | 0.069 | -0.007 | -0.091 – 0.077 | 0.859 |
| Observations | 57 |  |  | 31 |  |  |
| R^2^ / R^2^ adjusted | 0.080 / -0.010 | | | 0.320 / 0.184 | | |
| CIV: specific complex IV activity; CS: specific citrate synthase activity; *B:* regression coefficient (unstandardized); 95% CI: 95% confidence interval of the regression coefficient. Significant *P*-values are in bold. Nominal *P-*values are given. | | | | | | |

| **Supplementary Table 3: Multiple linear regression models of enzymatic activity of CS, CI, and CIV in platelet samples from PwPs and controls, without smokers.** | | | | | | |
| --- | --- | --- | --- | --- | --- | --- |
|  | *Dependent variable* | | | | | |
|  | **log(CI/CS)** | | | **log(CIV/CS)** | | |
| Predictors | *B* | 95% CI | *P*-value | *B* | 95% CI | *P*-value |
| Status (PD) | -0.007 | -0.086 – 0.072 | 0.855 | -0.026 | -0.071 – 0.020 | 0.267 |
| Age | 0.002 | -0.003 – 0.006 | 0.470 | 0.003 | 0.001 – 0.006 | **0.008** |
| Sex (Male) | 0.002 | -0.069 – 0.073 | 0.958 | -0.005 | -0.049 – 0.039 | 0.832 |
| Batch 1 | Ref. | - | - | - | - | - |
| Batch 2 | 0.044 | -0.045 – 0.134 | 0.323 | -0.004 | -0.055 – 0.048 | 0.892 |
| Batch 3 | -0.121 | -0.197 – -0.044 | **0.003** | -0.047 | -0.099 – 0.005 | **0.075** |
| Observations | 61 | | | 84 | | |
| R^2^ / R^2^ adjusted | 0.241 / 0.172 | | | 0.123 / 0.067 | | |
| CI: specific complex I activity; CIV: specific complex IV activity; CS: specific citrate synthase activity; *B:* regression coefficient (unstandardized); 95% CI: 95% confidence interval of the regression coefficient. Significant *P*-values are in bold. Nominal *P-*values are given. | | | | | | |

| **Supplementary Table 4. Multiple linear regression models of CI and CIV activity in the PD group.** | | | | | | |
| --- | --- | --- | --- | --- | --- | --- |
|  | *Dependent variable* | | | | | |
|  | **log(CI/CS)** | | | **log(CIV/CS)** | | |
| *Predictors* | *B* | 95 % CI | *P*-value | *Estimates* | 95% CI | *P*-value |
| Age | 7.2e-05 | -0.005 – 0.006 | 0.979 | 0.003 | -0.001 – 0.006 | 0.150 |
| Sex (Male) | -0.003 | -0.084 – 0.077 | 0.931 | -0.001 | -0.054 – 0.051 | 0.964 |
| Disease duration (months) | -2.9e-05 | -0.001 – 0.001 | 0.956 | -4.6e-04 | -0.001 – 0.000 | 0.102 |
| MDS-UPDRS III score | 0.002 | -0.002 – 0.006 | 0.278 | -0.001 | -0.003 – 0.001 | 0.359 |
| Batch 1 | Ref. | - | - | - | - | - |
| Batch 2 | 0.025 | -0.073 – 0.123 | 0.603 | -0.006 | -0.074 – 0.062 | 0.858 |
| Batch 3 | -0.116 | -0.213 – -0.019 | **0.020** | -0.065 | -0.133 – 0.003 | 0.062 |
| Observations | 40 | | | 54 | | |
| R^2^ / R^2^ adjusted | 0.306 / 0.180 | | | 0.177 / 0.072 | | |
| CI: complex I activity; CIV: complex IV activity; CS: citrate synthase activity; *B:* regression coefficient (unstandardized); 95% CI: 95% confidence interval of the regression coefficient; MDS-UPDRS III score: sum of MDS-UPDRS part III; Disease duration (months), duration of motor symptoms in months. Significant *P-*values are in bold. Nominal *P-*values are given. | | | | | | |
